# Transcriptional Profiles from Paired Normal Samples Offer Complementary Information on Cancer Patient Survival – Evidence from TCGA Pan-Cancer Data

**DOI:** 10.1038/srep20567

**Published:** 2016-02-03

**Authors:** Xiu Huang, David F. Stern, Hongyu Zhao

**Affiliations:** 1 Program in Computational Biology and Bioinformatics, Yale University, New Haven, CT 06520, USA; 2 Department of Pathology, School of Medicine, Yale University, New Haven, CT 06520, USA; 3 Department of Biostatistics, Yale School of Public Health, New Haven, CT 06520, USA

## Abstract

Although normal tissue samples adjacent to tumors are sometimes collected from patients in cancer studies, they are often used as normal controls to identify genes differentially expressed between tumor and normal samples. However, it is in general more difficult to obtain and clearly define paired normal samples, and whether these samples should be treated as “normal” due to their close proximity to tumors. In this article, by analyzing the accrued data in The Cancer Genome Atlas (TCGA), we show the surprising results that the paired normal samples are in general more informative on patient survival than tumors. Different lines of evidence suggest that this is likely due to tumor micro-environment instead of tumor cell contamination or field cancerization effect. Pathway analyses suggest that tumor micro-environment may play an important role in cancer patient survival either by boosting the adjacent metabolism or the *in situ* immunization. Our results suggest the potential benefit of collecting and profiling matched normal tissues to gain more insights on disease etiology and patient progression.

Although it is common practice to collect germ line information from cancer patients to identify somatic mutations in whole exome sequencing or whole genome sequencing (WGS) studies, it is much less common to have matched normal tissues from cancer patients for comparative gene expression studies. This is due to several reasons. Firstly, normal controls are not always available since the legitimate normal control for solid tumors should be collected from tissues residing near the tumor tissue site, which is harder to obtain than blood samples that can be used for germ line DNA sequence analysis. As a result, most solid tumor studies include no or only limited paired normal samples. Secondly, there is the concern of whether histologically-normal samples are truly ‘normal’ or they are actually in an abnormal state bearing genomic and translational aberrations corresponding to tumor, leading to potential biases in contrasting tumor to paired normal samples^1^,^2^. As a result, paired normal samples are rarely used in cancer genomics studies. For example, only tumor samples are used to define cancer subtypes and predict cancer outcomes, such as those for breast cancer PAM50, a widely adopted breast cancer signature panel that was developed based solely on gene expression profiles in tumor samples^3^. Essentially all progress in cancer genomics, such as subtyping and prognosis prediction, has been based on the analysis of tumor samples and the foreknowledge of candidate genes based on cancer genetics and pathways.

Although the value of paired normal samples in solid tumors has not been carefully examined, recent reports suggest that expression level changes between tumor and paired normal samples may be more correlated with cancer relapse and survival than expression levels in tumor samples alone^4^,^5^. Other studies have found that gene expression levels in normal tissues are more predictive of patient survival than tumor samples^6^,^7^. These studies suggest that normal samples may offer useful information to predict disease prognosis. There are several reasons that may explain the information in paired normal samples: 1) tumor cell contamination theory^8^, which was originally proposed to explain the high local recurrence rates of breast cancer after surgery, resulting from tumor cells extending beyond the invasive tumor margin and leading to genomic and translational signals in paired normal tissues; 2) field cancerization theory^1^, which was proposed to explain the multifocality of primary tumors, suggesting that paired normal tissues are in an intermediate state between normal and tumor, thus bearing information on early tumor initialization and development; and 3) tumor microenvironment theory^9^, which was proposed to account for the aberrant signals observed in patients’ extracellular matrix and non-malignant cells compared with those of non-patients, suggesting that normal tissues contain information about microenvironment surrounding tumors that either promotes or suppresses tumor development. These three theories may explain the observed signals on cancer progression in the histologically normal tissues. The field cancerization and tumor microenvironment theories in fact suggest that the signals in paired normal samples do provide additional information beyond what is offered in tumor tissues. However, there has not been a systematic study on the extent to which paired normal samples offer information on cancer subtyping and prognosis in different cancer types.

In this study, using the large datasets from multiple cancer cohorts collected by The Cancer Genome Atlas (TCGA)^10^ (http://cancergenome.nih.gov) with paired tumor and normal samples, we performed a comprehensive evaluation on whether and how much the paired normal samples may contribute to survival prediction. In particular, we aim to answer the following three questions: 1) in terms of prognosis signal, do paired normal samples offer additional information on patient prognosis beyond what can be explained by tumor cell contamination? 2) Across cancer types, how much benefit can we get from incorporating expression profiles from paired normal samples into patients’ survival prediction, and how much does the benefit vary across different cancer types? and 3) Do we have enough evidence to support or refute the three competing theories discussed above and what biological insights can be learned from analyzing paired normal samples?

## Results

### Paired normal samples offer additional and biologically meaningful information on patient clustering

**Figure 1:**
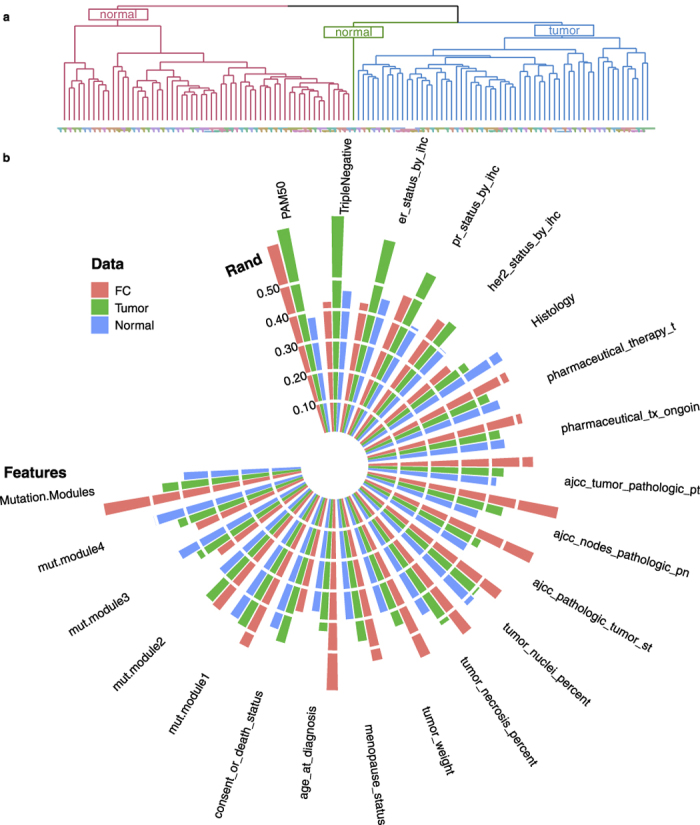
Adjacent normal samples’ transcriptional profiles offer different but clinically relevant information. (**a**) Hierarchical clustering of tumor and normal samples of 60 breast cancer patients with 6,000 most varying genes. (**b**) Rand indexes comparing groupings based on clinical or genetic features with those based on gene expression data with the 6,000 most varying genes of each data type using k-means clustering with k = 5, where a higher Rand index value indicates a higher degree of similarity.

To answer the first question, we first considered the TCGA breast cancer data where 60 pairs of matched tumor and normal tissue samples were gene expression profiled using the UNC Agilent G4502A_07 microarrays. We investigated whether normal tissue samples carry additional information on tumor prognosis in addition to that from the tumor tissues. We performed hierarchical clustering on the paired tumor-normal samples with 12,000 most varying genes. There is clear clustering of two distinct groups based on tumor and normal tissues (see Fig. 1a). We then clustered patients based on expression profiles from tumor samples, those from normal samples, and those defined by the tumor to normal fold changes, respectively, resulting in three different sample clusters. We then evaluated clustering consistency across these three separate clusters using Backer’s Gamma index to summarize the similarities between clusters. We also varied the number of genes used in clustering and the results are shown in Supplementary Table S1. It can be seen that tumor and normal samples have distinct clustering patterns for these patients, regardless the number of genes used for clustering. As the fold change levels were derived from tumor and normal data, clusters based on fold changes were similar to those either derived from tumor samples or those derived from normal samples, respectively. This result suggests that normal samples’ transcriptional signals are different from those of tumor samples.

We then investigated the biological relevance of the clusters derived from tumor samples, normal samples, and fold changes between the tumor and normal samples by correlating these clusters with clinical information from the patients. For these 60 patients, we extracted 17 different types of clinical features from the TCGA database. These clinical features include molecular features originally defined in tumor tissues, such as PAM50 and TripleNegative; they also include survival and patient status related features, such as histology and tumor weight. A detailed list of the clinical features is shown in Supplementary Table S2. We then used the Rand index to quantify the similarity of the groupings between sample clusters from gene expression levels/changes and clinical features, and evaluated the statistical significance of the similarity through permutation analysis. We formed sample clusters from gene expression data using the K-means method where K was set to be 5 (for other *k* values see Supplementary Fig. S2). The results are shown as the top 17 features of Fig. 1b. We found that, for molecular features, clusters derived from tumor samples had higher concordance with these features. These molecular level clinical features include hormone receptor status and also breast cancer subtypes, such as PAM50 and triple negative. This is not surprising because hormone receptor status and PAM50 subtypes are related to or derived directly from gene expression data from tumor tissues. On the other hand, there is better concordance between sample clusters defined from normal samples or fold changes and other clinical features, especially for survival related features, such as histology, tumor stage, tumor weight, and age at diagnosis. This suggests that not only do normal samples provide distinct information from that of the tumor samples, but they may also provide information that is more relevant to patient survival. It is unlikely that such information is derived from tumor cell contaminations in the normal tissues.

Besides associations with clinical features, we also explored how patient clustering is related to tumorigenesis and cancer progression. Cancer onset and progression result from the accumulation of somatic mutations on genes in the cancer-driving pathways. Mutual exclusivity refers to the observation that somatic mutations are often only observed in one of multiple genes in the cancer driving pathways^11^. Leiserson and colleagues^12^ used the same TCGA breast cancer cohort to identify driver pathways and identified four pathway modules showing mutually exclusive mutation patterns. A detailed list of the four gene set modules is shown in Supplementary Table S3. For each of the four gene set modules, we can use the exome sequencing data from the tumors to cluster the patients based on the mutation status for genes in this module. This resulted in four new features for these patients. These are represented as the 18th, 19th, 20th and 21st features in Fig. 1b. We then evaluated the concordance between these four new features and cancer patient clusters derived from tumor samples, paired normal samples, and fold changes between tumor and normal samples, respectively. The results in Fig. 1b show that except for the second module, the other three modules all have a better concordance with the groupings from either fold change or normal data than tumor data, suggesting that the information in normal data is also in some way related to the mutation status in the mutually exclusive components in the driver pathways. Furthermore, we grouped patients into 16 categories based on the joint mutation status at the module level, where we hypothesize that patients with different modules mutated may have different tumor genesis and cancer development mechanisms. For each module, we consider this module mutated if any gene in this module is mutated. By labeling each module as “1” or “0” depending on whether this module is mutated or not, each patient can be assigned to one of the 2^4^ = 16 different categories. The number of patients belonging to each category is listed in Supplementary Table S4. We studied the concordance between these 16 categories and sample clusters derived from gene expression data from tumor, normal or fold change data and the results are shown as the last feature of Fig. 1b. We can see that generally fold change data and normal data have higher concordance with the joint module mutation patterns. We further considered the somatic mutations in five key cancer driver genes in the tumor samples and adjacent normal samples of these 60 patients. The results shown in Supplementary Table S5 suggest that adjacent normal samples tend to have many fewer hits than tumor samples, and the somatic mutation profiles in tumors are distinct from adjacent normals. Both tumor and normal samples bear unique somatic mutations, with key tumor driving mutations preferentially found in tumor samples. Therefore, it is less likely that the survival related information in normal samples is due to the early development of cancer driving mutations, which is the key assumption in the field cancerization theory. This observation also contradicts expectation from the contamination theory.

### Incorporating expression profiles from paired normal tissues can improve cancer patient survival predictions across multiple cancer cohorts

**Figure 2:**
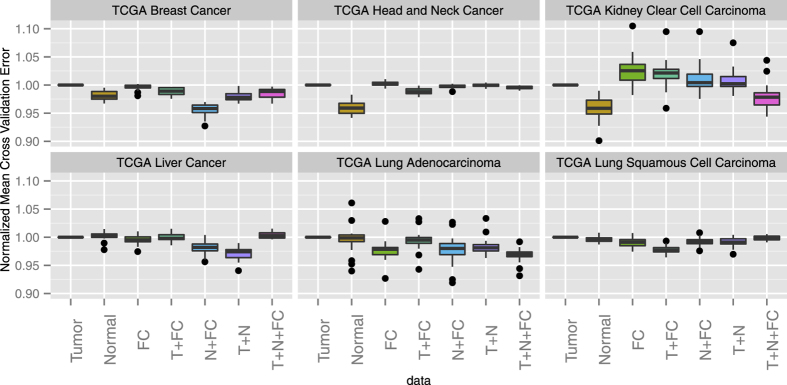
Normal samples consistently offer additional information to improve survival prediction performances across multiple cancer cohorts. The figures show the boxplots comparing distributions of survival prediction Mean Cross Validation Error from 20 random runs for penalized cox regression model from different datasets for six cancer cohorts of the RNASeq data.

To directly assess the usefulness of gene expression data from paired normal tissues in predicting patients’ survival and to further generalize to other platforms and cancer cohorts, we performed survival analysis on the TCGA pan-cancer (PANCAN) data where IlluminaHiSeq_RNASeqV2 was used to measure gene expression levels. There are a total of 12 cancer cohorts with matched tumor and normal sample pairs. We removed samples with incomplete survival information and cancer cohorts with insufficient number of matched normal samples, resulting in six cancer cohorts with sufficient numbers of samples and events (death). Table 1 shows the number of tumor-normal pairs and the number of cases available in these six cancer cohorts. For each of these six cancer cohorts, we compared the survival prediction accuracy using expression data from the tumor samples, the normal samples, and the fold changes between tumor and normal samples, respectively. We further considered four concatenated datasets using a combination of either two (tumor + normal, tumor + fold change, normal + fold change) or all three data types to explore the benefit of incorporating different data sources to improve prediction. We applied two survival analysis methods to ensure that the general conclusion does not depend on the specific statistical method used for cancer survival prediction. The results based on the penalized Cox regression survival analysis are shown in Fig. 2, and the results from the random forests survival analysis are shown in Supplementary Fig. S3. We can see that incorporating expression data from paired normal samples, either by directly using, contrasting or concatenating with tumor data, can always improve prediction accuracy. Specifically, for breast cancer, head and neck cancer and kidney clear cell carcinoma, expression data from paired normal samples can provide better prediction than tumor samples; whereas for liver cancer, lung adenocarcinoma, and lung squamous cell carcinoma, fold changes provided the most accurate predictions than either tumor samples or normal samples alone. The results from the random forests survival analysis were similar. Note that whether normal samples are more or less informative than tumor samples on survival does not correspond to the local recurrence rate of the specific cancer cohorts. For example, the recurrence rate of lung adenocarcinoma is generally higher than that of breast cancer^13^. However, paired normal samples for lung adenocarcinoma are less informative than those of breast cancer, suggesting that the signals in normal samples are unlikely due to the field cancerization effect, if we assume that the local recurrence rate is indicative of the surrounding tissue’s field cancerization level. Also, the results show that more information does not necessarily give better prediction result. It is possibly due to the inadequacies in the survival analysis methodologies to deal with high dimensional data. In fact, simple concatenation might not be an effective way to incorporate information from different data types in this context.

**Figure 3:**
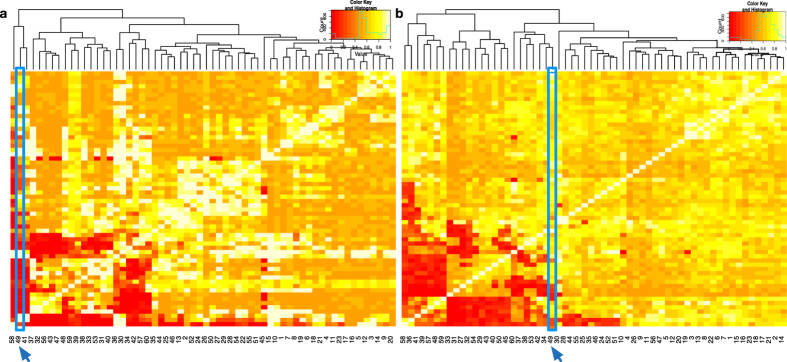
Normal samples provide an overall better prediction accuracy for patient survival and can be especially helpful for those patients with outlying survival prediction performances using tumor sample data alone. (**a**) Heatmap of the consistency matrix from tumor samples in the 60-patient breast cancer data using Elastic Net Cox Regression. Red color represents low consistency, while yellow represents high consistency. Individual 49 is highlighted with blue arrow as being an outlier with generally poor consistency of prediction with real survival using tumor data. (**b**) Consistency matrix calculated from correspondent normal data. Individual 49 is highlighted also, and is generally with comparable consistency levels with others individuals and no longer outlying.

We also applied the Elastic Net Regularized Cox Regression model directly in the survival prediction scheme to assess survival related information contained in adjacent tumor samples in the microarray platform on the 60 patients breast cancer data set. For each pair of samples, we calculated the relative risk predictions from the model built using the rest of samples. The consistency matrix *C* is then defined as the difference between the probability matrix of the relative risk comparison between each pair of samples and the correspondent probability matrix derived from the observed survival data. This matrix is informative as to suggest how the data perform in terms of survival prediction for every pair of samples. Each entry of the consistency matrix *C* is the consistency score of the predicted relative risk status for the corresponding two samples compared with the relative risk status from the real survival data. The heatmap plots of the matrix *C* for tumor data and normal data are shown in Fig. 3. Here the *α* parameter for the Elastic Net Regularized Cox Regression is set at 0.01, and the *β* parameter is chosen by five fold cross validation. It can be seen from the plot that the consistency level with the observed survival information from normal data is generally higher than that from tumor data. In the left panel, the blue arrow indicates an outlying sample with generally very low consistency in terms of survival prediction using tumor data. While the same patient is also highlighted with blue arrow in the right panel, with normal data, this sample’s performance in terms of survival prediction consistency is generally higher, and not very outlying compared with other samples. This result suggests that with the availability of paired normal samples, we can potentially elevate the overall survival prediction accuracy, especially for those patients with very poor and outlying survival prediction performance using only tumor tissue data.

### Immune, metabolic and cell growth related pathways in the paired normal samples are predictive of patient survival across various cancer cohorts

**Figure 4:**
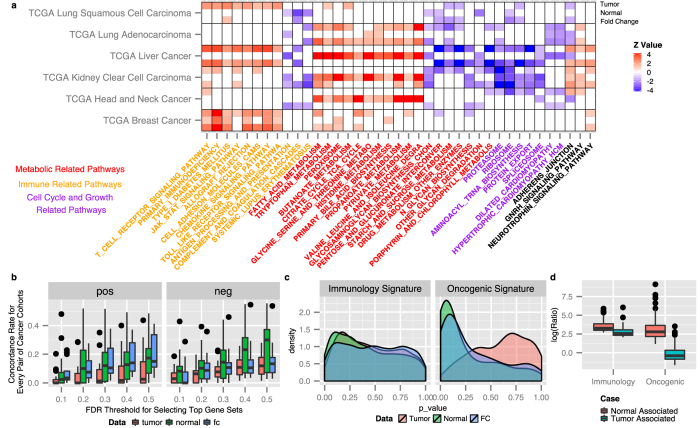
Gene set analyses show that metabolic, immune and cell growth related pathways are involved in boosting the survival related signals in adjacent normal samples. (**a**) Heat map showing the enriched pathways with FDR less than 0.25 consistently found at least five times across 18 cancer cohorts by data types considerations. Cells are colored according to the enrichment level calculated by transforming FDR p values to z values. The red color shows pathways that are up regulated for longer-surviving patients, while the blue color shows pathways that are down regulated for longer-surviving patients. Each cell has three sub cells, representing tumor data, normal data, and fold change data respectively from up to down. X-axis labels are colored according to the pathway types, with red being metabolic related pathways, yellow being immune related pathways, and purple being cell growth related pathways. (**b**) Boxplots comparing the distribution of concordance rates for enriched pathways found for each pair of cancer cohorts using different data types and different FDR thresholds to select enriched pathways. (**c**) Density plots of Univariate Cox Regression p values (representing each gene set signature’s correlation with survival using different types of data) for the 60-patient breast cancer data. (**d**) Boxplots showing the distributions of the top 100 log ratios of the p values from Univariate Cox Regression between either normal vs. tumor (tumor associated) or tumor vs. normal (normal associated) for each gene set signature. This is also for the 60-patient breast cancer data set.

We used the gene set enrichment analysis (GSEA) method to identify biological pathways that are enriched for genes informative for cancer patient survival using different data sets (tumor samples, paired normal samples, or fold changes), and looked for shared pathways across cancer cohorts. We hypothesize that if there is a general mechanism driving the normal tissues to offer additional information on tumor prognosis in all the cancer cohorts, pathway analysis may capture consistent changes across cancer cohorts and shed lights on this mechanism. Figure 4a shows the pathways that were found to be enriched for genes informative for cancer survival at the FDR cut-off of 0.25 at least five times across 18 analyses (6 each cancer cohorts × 3 data types). For each significant cohort-sample combination, we calculated the enrichment level as the z value transformed from the FDR corrected p value generated by GSEA, and it is represented by the shade of the color in Fig. 4a, with red corresponding positive values (i.e. higher expression levels lead to longer patient survival) and blue corresponding to negative values (i.e. high expression levels lead to poorer patient survival). The most frequently found pathway types included metabolism related pathways, immune related pathways and cell cycle and growth related pathways, which are colored red, yellow and purple, respectively, in this figure. There is a clear enrichment of immune related pathways in the up regulated gene sets, whereas there is an enrichment of cell cycle related pathways in the down regulated gene sets. There are both enrichment hits of up regulated and down regulated gene sets related metabolism. This suggests that across cancer types, patients with increased immune activities and reduced cellular growth activities, either in tumor tissues, paired normal tissues, or fold changes between tumor and normal tissues, tend to have better prognosis. To investigate how different data types (tumor, normal, and fold change) contribute most to the consistent pathways found among cancer cohorts, we calculated the overall concordance between any two pairs of cancer cohorts for gene sets positively correlated with survival and gene sets negatively correlated with survival and plot the boxplot of concordance rates against varying FDR thresholds that were used to select the top pathways (see Fig. 4b). We can see that in gene sets positively correlated with survival, consistency is generally higher in fold change data and normal data, and for gene sets negatively correlated with survival, consistency is much higher in normal data, suggesting that the activation of immune related pathways and down regulation of cellular growth pathways in surrounding normal tissue environment is associated with longer survival. This pattern is found to be shared across all cancer cohorts studied here. Nevertheless, we do observe distinct patterns of pathways being hit for different cancer cohorts. For example, immune related pathways are more activated for breast cancer patients to live longer while cell cycle related pathways are more suppressed for liver cancer patients and kidney clear cell carcinoma patients to live longer.

To further examine the involvement of immune related functions and oncogenic related functions driving the survival related information in tumor and normal samples using an independent data set, we conducted individualized GSEA using the 60-sample breast cancer data set. For each individual sample, the signals from all the genes are summarized at the gene set level using individualized GSEA method. For each gene set, we used the gene set level perturbation values across patients to correlate with patient survival information using Univariate Cox Regression model. This analysis directly shows how the gene set perturbation levels for each pre-defined immune related and oncogenic related gene set signature are related to survival. Figure 4c shows the density plots of the distributions of the significance p values from Univariate Cox Regression model for all the immune signatures and oncogenic signatures for each data set. As for comparisons between tumor and normal samples, both immune signatures and oncogenic signatures show a more enriched concentration of p values towards zero for normal samples than tumor samples, although the enrichment for immune signatures is moderate while the enrichment for oncogenic signatures is more dramatic. This is consistent with what we found previously in the multi-cancer analysis, that the oncogenic signatures and the immune signatures are more predictive of survival in normal than in tumor samples. Also surprisingly, in tumor samples the immune signatures are more predictive of survival than oncogenic signatures.

To identify the top differentially performing pathways between tumor and normal tissues, we calculated the ratios of p values from the Cox model between normal and tumor data for each gene set, and identified the top 100 gene sets that are mostly differentiatedly related to survival between tumor and normal samples. The distributions of the log ratios is shown in Fig. 4d. We defined the top gene sets as either ‘Normal Associated’ or ‘Tumor Associated’ indicating whether this pathway is more predictive in normal or in tumor samples. We can see that although the overall enrichment of normal associated gene sets is moderate compared with tumor, the differentiated survival-associated gene sets are dramatically enriched, suggesting that more gene sets are reliably and specifically predictive of survival in normal than in tumor samples. As expected, this trend is more evident for oncogenic signature gene sets. This result suggests that both immune and oncogenic functions are relevant in survival related information in adjacent normal samples. The detailed table of the top 100 ‘normal associated’ and ‘tumor associated’ gene sets for the two signature categories is shown in Supplementary Table S6.

## Discussion

In this report, we have systematically evaluated whether transcriptional profiles of tumor adjacent normal samples are predictive of patient survival across multiple cancer cohorts and different platforms using the TCGA data. The results show that adjacent normal samples’ transcriptional level signals likely provide more information than tumor samples on patient survival across cancer types and data collection platforms. This result implies that the tumor surrounding tissues may harbor meaningful signals for cancer biogenesis and prognosis. This phenomenon could possible be explained by the idea of “etiologic field effect” proposed in a recent paper^14^, which is an extension of the traditional field cancerization theory^1^ combined with the tumor microenvironment theory^9^. This concept gives a coherent model to explain the signals in the adjacent normal tissues by promoting the idea of the tissue microenvironment milieu with aberrant signals in either the genome, epigenome, transcriptome, proteome, metabolome or interactome that promotes the behavior of both the tumor and the host immunity.

This finding could further our understanding of cancer etiology and better identify biomarkers for more accurate cancer diagnosis and prognosis, as well as personalized medicine and treatment design. The collection and molecular characterization of adjacent normal tissues, in addition to tumor tissues, may allow researchers to gain more insights on disease etiology and patient progression. The contribution of paired normal samples in prognosis prediction can be interpreted as the contribution of “tumorigenesis code” carried by the normal tissues. This code may represent the patient’s overall immunity or metabolic level, which may lead to more effective personalized medicine. These results also suggest that we may better understand tumorigenesis mechanisms and identify potential therapeutic targets through a more thorough exploration of surrounding microenvironment.

There are several limitations in our study. Firstly, there are no clear definitions for adjacent normal tissues. For the TCGA data set, the normal tissues were collected with an approximate 2 cm or 3 cm distance from the tumor margin. However, how the survival related information in normal tissues varied accordingly to the distance of normal towards tumor tissues needs to be assessed. The survival related information is of various relevance across cancer types, likely due to different tissue features and how the adjacent normal tissues were obtained. More needs to be done to adjust for the effects from these factors. Secondly, adjacent normal tissues bear the neighboring microenvironment signals. It is of potential interest to consider the distal environment signals’ contribution towards tumor progression. There are publications already discussing the possibilities of finding marker genes in the circulating blood tissues^15^. Three-way comparisons of transcriptional signals in tumor, adjacent normal, and blood tissues would be of great interest to detect possible survival related signals shared between adjacent and distal microenvironment if such data sets are available.

## Methods

### Overview

We applied unsupervised clustering on the tumor and normal samples, as well as gene expression fold changes to match with groupings using clinical variables to assess whether normal samples offer clinically relevant information. We used Cox based and Random Forests^16^ based survival analysis to evaluate the information in gene expression data on patient survival. To identify the biological perturbations that are shared among different cancer cohorts, we conducted Gene Set Enrichment Analysis (GSEA)^17^ to identify gene sets that are informative on patient survival and these gene sets may lead to insights on the underlying biological processes informative on patient survival. An illustration of the detailed analysis workflow in correspondence to the three research questions is shown in Supplementary Fig. S1.

### Data sets

Transcriptome data and clinical data were obtained from the TCGA Data Portal (https://tcga-data.nci.nih.gov/tcga/tcgaHome2.jsp). We downloaded data in the category of UNC Agilent G4502A 07 microarrays for breast cancer with 60 pairs of paired tumor and solid normal samples. We also downloaded data in the category of UNC IlluminaHiSeq RNASeqV2 for six cancer types with their paired tumor and normal samples. The numbers of pairs of tumor and normal samples for each cancer types are listed in Table 1.

### Unsupervised clustering of samples

#### Hierarchical clustering

Genes were ranked based on the Median Absolute Deviance (MAD) of expression levels and those genes with the largest MAD values were selected for clustering. We used Euclidean distance to measure the distance between genes and complete linkage for hierarchical clustering. Backer’s Gamma index^18^ was used to quantify the similarity between hierarchical trees constructed using tumor samples, those using normal samples and those using fold change values between tumor and normal samples. The statistical significance of the Backer’s Gamma index was assessed through 1000 permutations of the samples.

#### Correlating gene clusters with clinical features

For each data set, we used MAD values to select the top 6000 most varying genes for K-means clustering, where *k* was varied from 2–15. We performed k-means clustering on tumor samples, normal samples, and fold changes, respectively. We used the Rand index^19^ to measure the similarity between the clustering from gene expression and the groupings from available clinic features. We used 1000 permutations to estimate the significance of the Rand indexes.

### Survival analysis

Because of the high dimensionality of the data, we adopted the Elastic Net Penalized Cox Regression method^20^,^21^ and the nonparametric method Random Forests^16^,^22^ for survival analysis. Nonetheless, too many variables may still cause unnecessary noises and complexities in the model. Therefore, we employed a dimension reduction step to alleviate the problem. More specifically, we modeled the survival outcome as a function of the principal components so that the dimensionality of the dataset is reduced from the total number of genes to at most the number of samples. By using the principal components as variables instead of the genes, we are able to reduce the dataset dimension and at the same time preserve most of the variance across samples.

#### Penalized cox regression

The elastic net regularization^21^ uses with the following penalization on *β*, which is a linear combination of *L*_1_ penalty and *L*_2_ penalty:


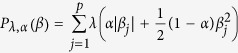


The tuning parameters *α* and *β* were selected by grid search from five fold cross validation. The goodness of fit is measured by mean cross validation error^20^, which is defined as:


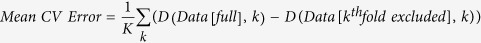


with *K* being the total number of folds, and with the *k*^*th*^ fold, *D*(*Data, k*) is defined as the residual sum of square deviance of real survival outcome versus predicted survival outcome using 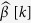, which is obtained from the *k*^*th*^ fold of data. The cross validation error of the *k*^*th*^ fold is defined as the difference between deviances evaluated on full data with that on data with the *k*^*th*^ fold excluded. We used the mean of each fold’s cross validation error to assess the goodness of fit.

We also applied this method in the context of survival prediction to assess the prediction accuracy directly. For each pair of samples, we calculated the relative risk predictions from the Cox model built using the rest of samples. The data features were dimension-reduced and orthogonalized using PCA beforehand. The probability matrix *M* is defined as 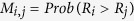 across 20 random runs, where *R*_*i*_ and *R*_*j*_ are the risk predictions of sample *i* and sample *j* from the Cox model derived using the rest of all the samples s: 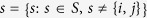 with five fold cross validation. The consistency matrix *C* is then defined as 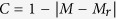, where *M*_*r*_ is the probability matrix derived from the real survival data. The entries of *M*_*r*_ are either 0 or 1 where the risk comparisons are identifiable for the specific sample pairs from the real survival data, and 0.5 anywhere else where the risk comparisons are uncertain because of the presence of censored data. The consistency matrix *C* is informative as to suggest how the data perform in terms of survival prediction.

#### Random Forests

In addition to penalized Cox regression, we used Random Forests^23^ to compare survival prediction accuracy from different data sources. We built 10,000 trees for Random Forests and used the out-of-bag error rate^24^ to measure prediction accuracy.

### Pathway analysis

To investigate the biological relevance of the signals in normal and tumor tissues, we performed pathway analysis to identify pathways predictive of survival outcome in various cancers. As input for pathway analysis, for each gene, we calculated the association between its gene expression levels and patient survival using Univariate Cox Regression model^25^, and the resulting p value transformed z value was used for pathway analysis. We adopted the Gene Set Enrichment Analysis (GSEA)^17^ method and used the Kyoto Encyclopedia of Genes and Genomes (KEGG)^26^ pathway database as the reference gene sets.

To further examine the relevance of immune signatures and oncogenic signatures with survival, we conducted individualized GSEA, which is to perform GSEA on each individual and get a summarization of gene level signal to pathway level signal. For gene expression data set with P genes and N samples, for each sample n, we will get the enrichment level of m predefined gene set signatures. For these m gene sets, we will then perform Univariate Cox Regression to identify gene sets that are mostly predictive of survival. The immune signature and oncogenic signatures are downloaded from MSigDB^17^.

### Statistical analysis

All analyses were performed using the R programming platform^27^. All the plots are generated using R package ggplot2^28^.

## Additional Information

**How to cite this article**: Huang, X. *et al*. Transcriptional Profiles from Paired Normal Samples Offer Complementary Information on Cancer Patient Survival – Evidence from TCGA Pan-Cancer Data. *Sci. Rep.* **6**, 20567; doi: 10.1038/srep20567 (2016).

## Supplementary Material

Supplementary Information

## Figures and Tables

**Table 1:** Sample sizes for the six cancer cohorts in the TCGA multi-cancer RNA-Seq data.

Cancer Cohorts	Number of paired samples/Number of events (death)
Breast Cancer	105/37
Kidney clear cell carcinoma	70/24
Lung adenocarcinoma	57/22
Liver Cancer	46/29
Lung Squamous cell carcinoma	42/24
Head and neck cancer	40/31
