# Supplementary material for: Transcriptional Profiles from Paired Normal Samples Offer Complementary Information on Cancer Patient Survival – Evidence from TCGA Pan-Cancer Data
Source: Sci Rep. 2016 Feb 3;6:20567. doi: 10.1038/srep20567 (PMC4738355; doi:10.1038/srep20567)
Supplement: Supplementary Information [file srep20567-s1.pdf]

Transcriptional Profiles from Paired Normal Samples Offer Complementary Information on Cancer Patient Survival -- Evidence from TCGA Pan-Cancer Data

Xiu Huang, David Stern, and Hongyu Zhao

Supplementary Materials

Fig. S1. Diagram illustrating the analyses performed in this article.

Fig. S2. Rand indexes comparing groupings based on clinical or genetic features with those based on gene expression data of each data type.

Fig. S3. Boxplot comparing distributions of Out Of Bag Error Rate from 20 random runs for Random Forest survival model using different datasets for six cancer cohorts of the RNASeq data.

Fig. S4. Barplot of the similarity levels of the sample expression barcodes versus cell type specific barcodes defined by the gene expression barcode project.

Fig. S5. Barplot of the Rand indexes comparing stratifications using different types translational data and using clinical variables.

Fig. S6. Boxplot of the similarity levels of the sample expression barcodes versus cell type specific barcodes defined by the gene expression barcode project.

Table S1. Backer's Gamma index comparing grouping similarity of hierarchical clustering from each two of the tumor, normal and fold change data types.

Table S2. Detailed list of the clinical features' explanations and grouping structures.

Table S3. Detailed list of the four mutually exclusive gene set modules.

Table S4. Detailed list of the sample sizes of each category of patients according to the mutation patterns of the four driving modules in the 60 breast cancer patients' data.

Table S5. Number of patients bearing somatic mutations on key tumor driving genes in tumor samples, adjacent normal samples and both normal and tumor samples.

Table S6. Top 100 gene set signatures being differentially associated with survival.

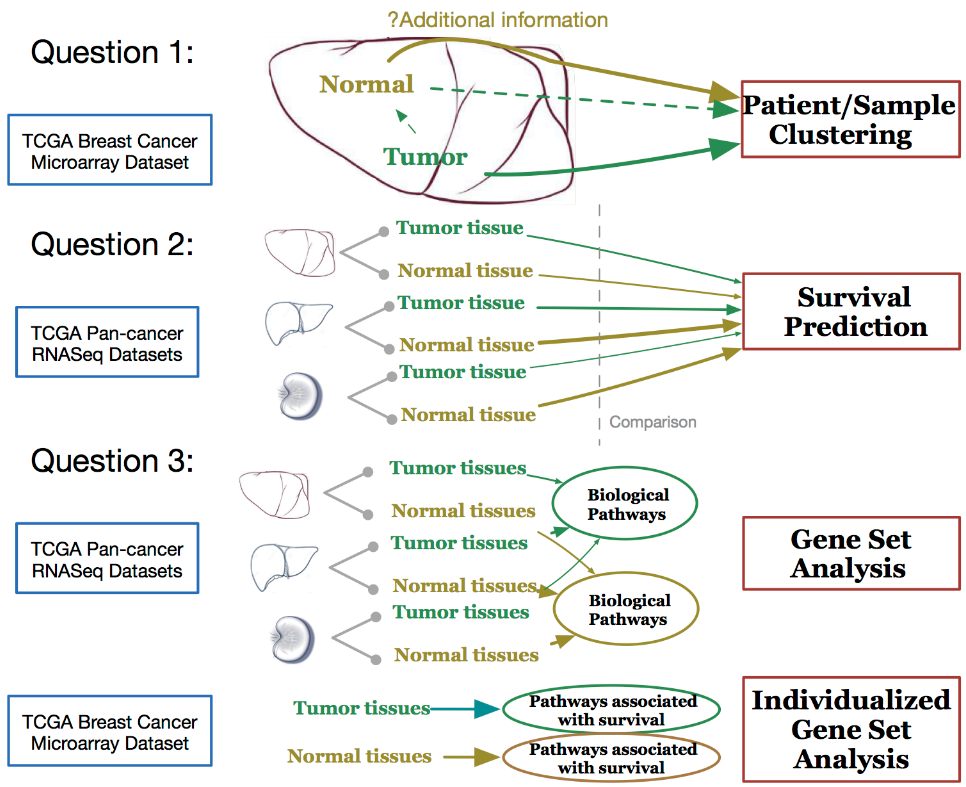

Fig. S1. Diagram illustrating the analyses performed in this article.

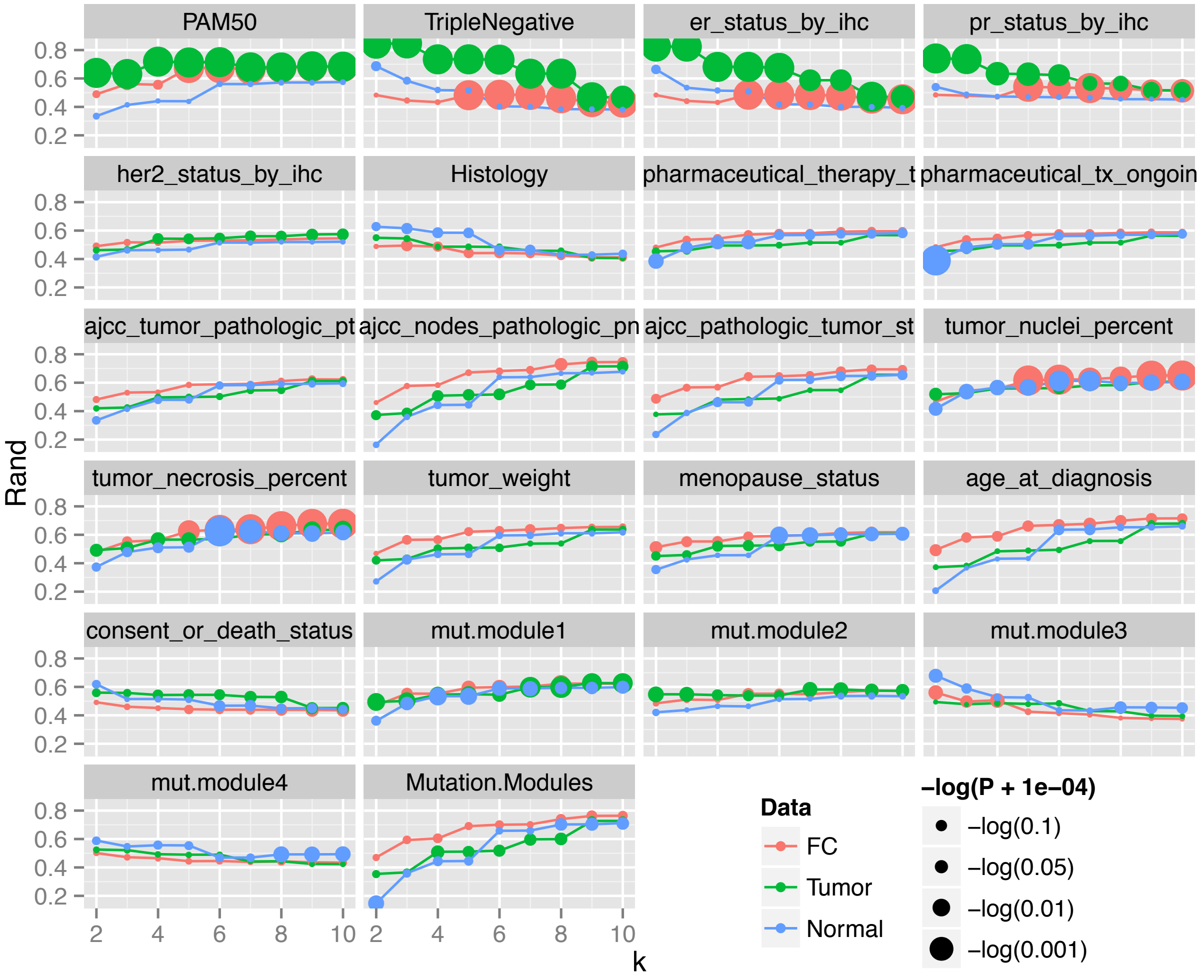

Fig. S2. Rand indexes comparing groupings based on clinical or genetic features with those based on gene expression data of each data type. In each panel, there are three curves representing the Rand index values for comparing clinical features with clusters derived from tumor samples (green), normal samples (blue), and expression level changes between tumor and normal samples (red) using 6,000 most varying genes, respectively. Different points of each curve correspond to clinical features clustered using k-means clustering where k is varied from 2 to 10. The statistical significance level of similarity is calculated from permutation and represented by the size of the filled circle.

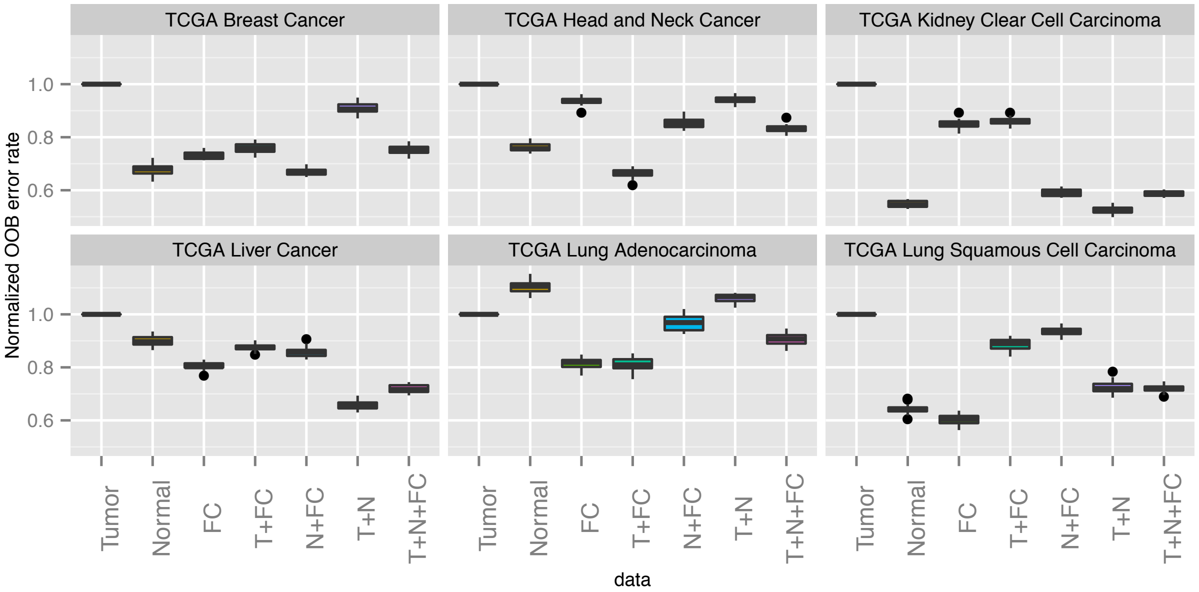

Fig. S3. Boxplot comparing distributions of Out Of Bag Error Rate from 20 random runs for Random Forest survival model using different datasets for six cancer cohorts of the RNASeq data.

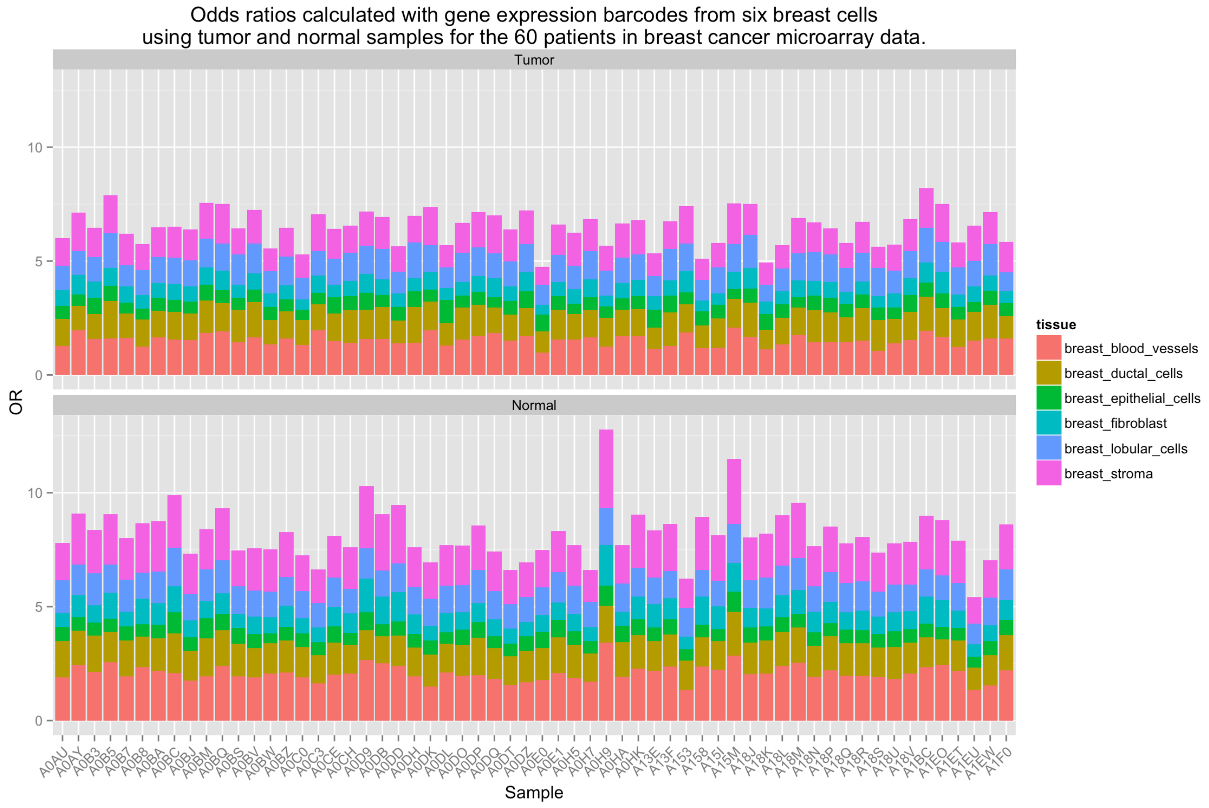

Fig. S4. Barplot of the similarity levels of the sample expression barcodes versus cell type specific barcodes defined by the gene expression barcode project.

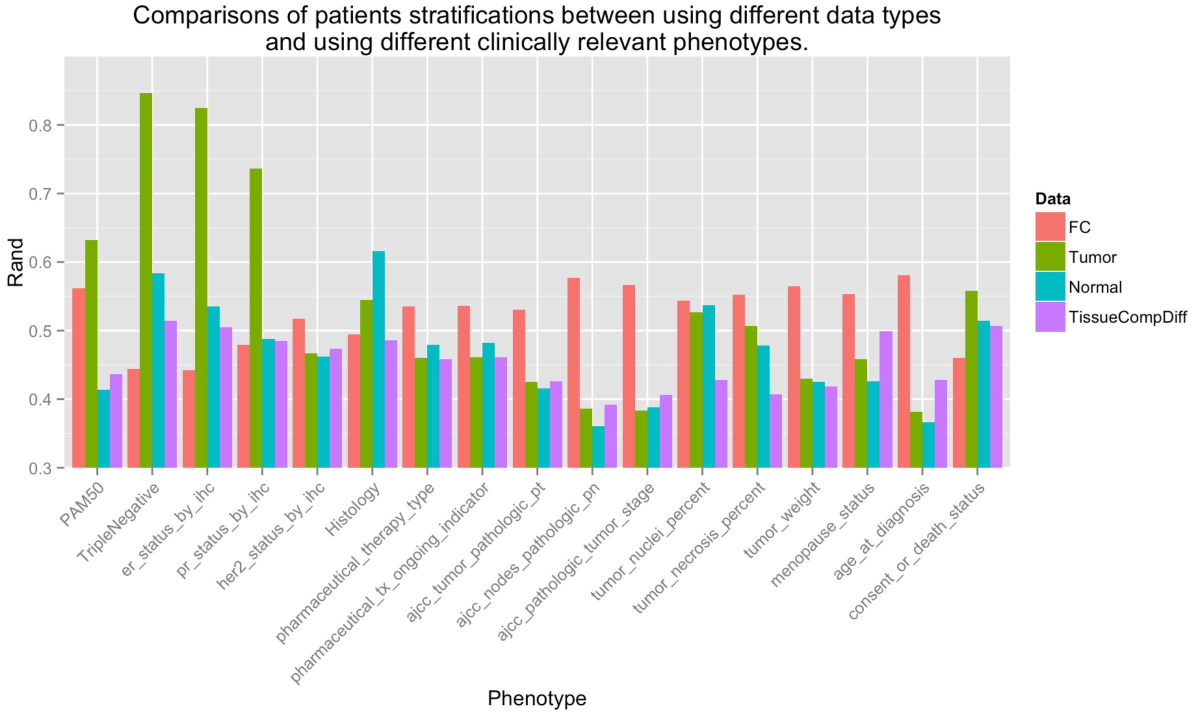

Fig. S5. Barplot of the Rand indexes comparing stratifications using different types of translational data and using clinical variables.

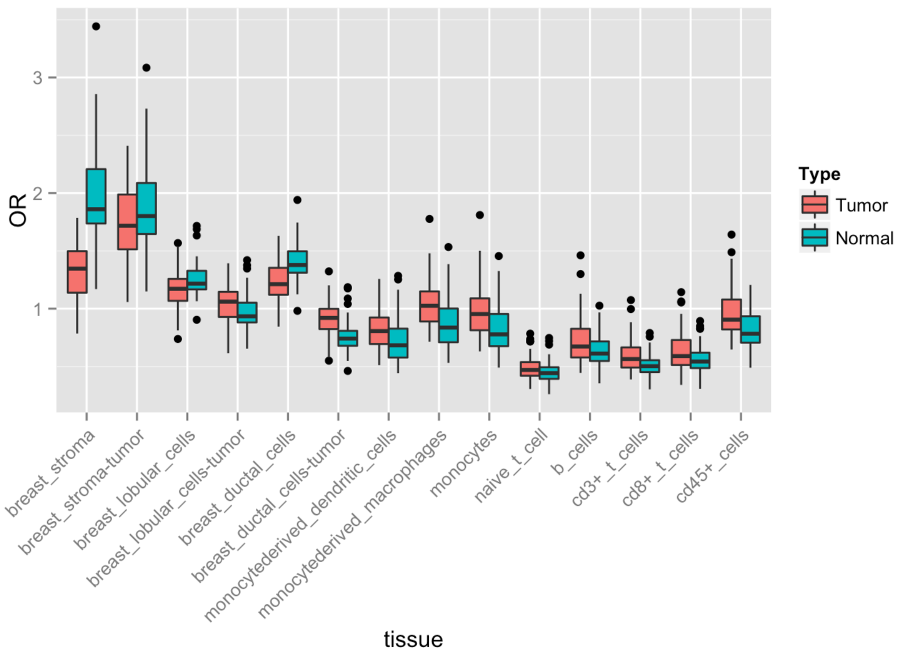

Fig. S6. Boxplot of the similarity levels of the sample expression barcodes versus cell type specific barcodes defined by the gene expression barcode project.

	fc vs. tumor	fc vs. normal	tumor vs. normal
**Top 1000 Genes**	0.13/0.04*	0.71/0*	-0.04/0.695
**Top 2000 Genes**	0.21/0.004*	0.31/0*	-0.03/0.644
**Top 3000 Genes**	0.09/0.105	0.28/0*	-0.06/0.79
**Top 4000 Genes**	0.16/0.021*	0.26/0*	-0.07/0.831
**Top 5000 Genes**	0.18/0.013*	0.26/0*	-0.03/0.644
**Top 6000 Genes**	0.14/0.032*	0.28/0*	-0.05/0.748
**Top 7000 Genes**	0.15/0.024*	0.23/0.002*	-0.03/0.644
**Top 8000 Genes**	0.43/0*	0.49/0*	-0.04/0.695
**Top 9000 Genes**	0.31/0*	0.3/0*	-0.03/0.644
**Top 10000 Genes**	0.43/0*	0.45/0*	-0.02/0.572
**Top 11000 Genes**	0.48/0*	0.47/0*	-0.01/0.521
**Top 12000 Genes**	0.12/0.054	0.69/0*	-0.05/0.748
**Top 13000 Genes**	0.22/0.002*	0.6/0*	-0.06/0.79
**Top 14000 Genes**	0.41/0*	0.47/0*	-0.04/0.695
**Top 15000 Genes**	0.24/0.002*	0.6/0*	-0.05/0.748
**Top 16000 Genes**	0.24/0.002*	0.62/0*	-0.05/0.748
**Top 17000 Genes**	0.24/0.002*	0.62/0*	-0.05/0.748
**Top 18000 Genes**	0.25/0.001*	0.62/0*	-0.05/0.748

Table S1. Backer's Gamma index comparing grouping similarity of hierarchical clustering from each two of the tumor, normal and fold change data types. Each entry in the table shows both the index value and the statistical significance of the similarity between the two clusters, i.e. smaller p-values indicating evidence suggesting similarity.

Clinical features	Explanation	Categorical sample size
PAM50	A 50-gene-expression signature.	10 Basal; 4 Her2; 31 LumA; 13 LumB, 2 NA.
TripleNegative	Breast cancer that does not express ER, PR and Her2.	8 TripleNegative; 50 Non-TripleNegative; 2 NA.
ER status	Estrogene receptor status by immunohistochemistry.	49 Positive; 9 Negative; 1 Indeterminate; 1 NA.
PR status	Progesterone receptor status by immunohistochemistry.	42 Positive; 16 Negative; 1 Indeterminate; 1 NA.
HER2 status	Human epidermal growth factor receptor 2 status by immunohistochemistry.	10 Positive; 37 Negative; 8 Equivocal; 5 NA.
Histology	Sample histological type.	48 Infiltrating ductal carcinoma; 4 Infiltrating Lobular Carcinoma; 1 Mucinous Carcinoma; 5 Mixed histology; 2 NA.
Pharmaceutical therapy type		11 Chemotherapy; 18 Hormone Therapy; 1 Chemotherapy or Hormone Therapy; 30 NA.
Pharmaceutical tx ongoing indicator	Indicator of whether the pharmaceutical treatment is still ongoing.	18 Yes; 12 No; 30 NA.
AJCC tumor pathologic type	Tumor pathologic type classified by the American Joint Committee on Cancer (AJCC) system.	4 T1; 1 T1a; 1 T1b; 14 T1c; 30 T2; 1 T2b; 8 T3; 1 T4b.
AJCC nodes pathologic type	Lymph nodes pathologic type classified by the American Joint Committee on Cancer (AJCC) system.	14 N0; 11 N0(i-); 7 N1; 13 N1a; 4 N1b; 2N1c; 2 N1mi; 1 N2; 3 N2a; 1 N3; 2 N3a.
AJCC pathologic tumor stage	Pathologic tumor stage classified by the American Joint Committee on Cancer (AJCC) system.	12 Stage I; 1 Stage IA; 19 Stage IIA; 16 Stage IIB; 8 Stage IIIA; 3 Stage IIIC; 1 Stage IV.
Tumor nuclei percent	Percentage of tumor cells in the sample.	2 60; 36 70; 3 75; 9 80; 3 85; 4 90; 3 95.
Tumor necrosis percent	Percentage of cell death in the sample.	13 0; 1 1; 2 2; 6 5; 4 10; 1 15; 33 30.
Tumor weight		11 (, 200); 25 [200,400); 15 [400,600); 3 [600,800); 4 [800,1000); 2 [1000,).
Menopause status		19 Pre; 1 Peri; 28 Post; 1

		Indeterminate; 11 NA.
Age at diagnosis		6 [0,40); 15 [40,50); 15 [50,60); 14 [60,70); 6 [70,80); 4 [80,).
Consent or death status		46 Consented; 14 Deceased.

Table S2. Detailed list of the clinical features considered and grouping structures. The clinical features were used to group the 60 breast cancer patients into subgroups and these subgroups were compared with the groupings from hierarchical clustering using transcriptional data of tumor samples, normal samples and fold changes respectively to evaluate the clinical relevance of transcriptional signals from each source.

Module Name	Genes in the module	Categorical sample size
Module 1	PTEN, AKT1, PIK3CA, PIK3R1, 12p3.33(A), HIF3A	2 with PTEN mutated; 2 with AKT1 mutated; 25 with PIK3CA mutated; 1 with PIK3R1 mutated; 2 with 12p3.33(A) mutated; 1 with 12p13.33(A) and HIF3A both mutated; 1 with PTEN and PIK3R1 both mutated; 26 with this module not mutated.
Module 2	TP53, CDH1, GATA3, CTCF, GPRIN2	16 with TP53 mutated; 2 with CDH1 mutated; 4 with GATA3 mutated; 1 with CTCF mutated; 1 with GPRIN2 mutated; 36 with this module not mutated.
Module 3	MAP2K4(D), MAP3K1, PPEF1, WWP2, SMARCA4	4 with MAP2K4(D) mutated; 6 with MAP3K1 mutated; 1 with WWP2 mutated; 1 with SMARCA4 mutated; 48 with this module not mutated.
Module 4	CCDN1(A), RB1, MAP2K4, GRID1	9 with CCDN1(A) mutated; 1 with RB1 mutated; 3 with MAP2K4 mutated; 1 with GRID1 mutated; 46 with this module not mutated.

Table S3. Detailed list of the four mutually exclusive gene set modules.

	0000	0001	0010	0011	0101	0110	0111
Number of patients	2	2	2	5	2	7	14
	1000	1001	1011	1101	1110	1111	
Number of patients	1	2	10	3	2	8	

Table S4. Detailed list of the sample size of each category of patients according to the mutation patterns of the four driving modules in the 60 breast cancer patients' data.

	Number of patients bearing tumor only mutations	Number of patients bearing adjacent normal only mutations	Number of patients bearing mutations in both tissues
PIK3CA	12	0	1
TP53	8	3	1
CDH1	4	0	0
MAP3K1	2	0	0
MAP2K4	2	0	2

Table S5. Number of patients bearing somatic mutations on key tumor driving genes in tumor samples, adjacent normal samples and both normal and tumor samples.

	Immune Signatures	
	Normal Associated	
Rank	GeneSet	p_Tumor/ p_Normal
1	GSE17721_LPS_VS_GARDIQUIMOD_2H_BMDM_UP	360
2	GSE17721_CTRL_VS_LPS_1H_BMDM_DN	291
3	GSE18148_CBFB_KO_VS_WT_TREG_DN	289
4	GSE17721_CTRL_VS_GARDIQUIMOD_4H_BMDM_DN	249
5	GSE1432_CTRL_VS_IFNG_1H_MICROGLIA_DN	248
6	GSE18791_CTRL_VS_NEWCASTLE_VIRUS_DC_2H_DN	217
7	GSE360_L_DONOVANI_VS_L_MAJOR_DC_UP	211
8	GSE22886_TH1_VS_TH2_12H_ACT_DN	165
9	GSE17721_CTRL_VS_LPS_4H_BMDM_DN	161
10	GSE27786_LSK_VS_ERYTHROBLAST_DN	159
11	GSE3982_CTRL_VS_LPS_1H_NEUTROPHIL_UP	150
12	GSE7460_TREG_VS_TCONV_ACT_WITH_TGFB_UP	146
13	GSE7460_TREG_VS_TCONV_ACT_UP	112
14	GSE17721_CTRL_VS_CPG_1H_BMDM_DN	110
15	GSE20715_0H_VS_24H_OZONE_LUNG_DN	108
16	GSE1460_DP_THYMOCYTE_VS_THYMIC_STROMAL_CELL_DN	84
17	GSE29614_DAY3_VS_DAY7_TIV_FLU_VACCINE_PBMC_UP	78
18	GSE29615_CTRL_VS_DAY3_LAIV_IFLU_VACCINE_PBMC_UP	69
19	GSE20366_EX_VIVO_VS_DEC205_CONVERSION_NAIVE_CD4_TCELL_DN	61
20	GSE18791_CTRL_VS_NEWCASTLE_VIRUS_DC_12H_DN	61
21	GSE20366_EX_VIVO_VS_DEC205_CONVERSION_DN	60
22	GSE17721_0.5H_VS_4H_LPS_BMDM_DN	55
23	GSE18791_CTRL_VS_NEWCASTLE_VIRUS_DC_10H_DN	54
24	GSE24081_CONTROLLER_VS_PROGRESSOR_HIV_SPECIFIC_CD8_TCELL_UP	53
25	GSE2826_WT_VS_XID_BCELL_DN	50
26	GSE13484_UNSTIM_VS_3H_YF17D_VACCINE_STIM_PBMC_DN	47
27	GSE13411_NAIVE_VS_SWITCHED_MEMORY_BCELL_UP	46
28	GSE11924_TH1_VS_TH2_CD4_TCELL_DN	45
29	GSE360_T_GONDII_VS_M_TUBERCULOSIS_MAC_DN	45
30	GSE7460_WT_VS_FOXP3_HET_ACT_WITH_TGFB_TCONV_DN	45
31	GSE37416_CTRL_VS_3H_F_TULARENSIS_LVS_NEUTROPHIL_DN	44
32	GSE9988_LOW_LPS_VS_CTRL_TREATED_MONOCYTE_UP	43
33	GSE29618_PRE_VS_DAY7_POST_LAIV_FLU_VACCINE_PDC_UP	42
34	GSE17974_IL4_AND_ANTI_IL12_VS_UNTREATED_1H_ACT_CD4_TC	41

	ELL_UP	
35	GSE17974_0H_VS_0.5H_IN_VITRO_ACT_CD4_TCELL_DN	39
36	GSE2706_R848_VS_R848_AND_LPS_8H_STIM_DC_DN	39
37	GSE17721_CTRL_VS_PAM3CSK4_6H_BMDM_DN	38
38	GSE17974_IL4_AND_ANTI_IL12_VS_UNTREATED_6H_ACT_CD4_TCELL_DN	38
39	GSE2706_2H_VS_8H_R848_STIM_DC_DN	38
40	GSE17721_CTRL_VS_POLYIC_1H_BMDM_DN	36
41	GSE17580_UNINFECTED_VS_S_MANSONI_INF_TREG_DN	35
42	GSE39820_CTRL_VS_IL1B_IL6_CD4_TCELL_DN	33
43	GSE17721_LPS_VS_POLYIC_2H_BMDM_UP	33
44	GSE2706_LPS_VS_R848_AND_LPS_8H_STIM_DC_DN	32
45	GSE9037_CTRL_VS_LPS_1H_STIM_IRAK4_KO_BMDM_DN	31
46	GSE20366_CD103_POS_VS_NEG_TREG_KLRG1NEG_DN	31
47	GSE13411_NAIVE_VS_IGM_MEMORY_BCELL_UP	30
48	GSE17721_CTRL_VS_GARDIQUIMOD_1H_BMDM_DN	30
49	GSE20366_TREG_VS_TCONV_UP	28
50	GSE360_L_MAJOR_VS_M_TUBERCULOSIS_MAC_DN	26
51	GSE17974_IL4_AND_ANTI_IL12_VS_UNTREATED_48H_ACT_CD4_TCELL_DN	26
52	GSE17721_PAM3CSK4_VS_GADIQUIMOD_1H_BMDM_UP	26
53	GSE27786_LIN_NEG_VS_ERYTHROBLAST_DN	25
54	GSE22886_CD8_VS_CD4_NAIVE_TCELL_UP	25
55	GSE36392_TYPE_2_MYELOID_VS_MAC_IL25_TREATED_LUNG_UP	25
56	GSE2706_R848_VS_R848_AND_LPS_2H_STIM_DC_DN	25
57	GSE360_CTRL_VS_B_MALAYI_LOW_DOSE_DC_UP	25
58	GSE7460_FOXP3_MUT_VS_WT_ACT_WITH_TGFB_TCONV_UP	25
59	GSE7460_CTRL_VS_FOXP3_OVEREXPR_TCONV_DN	24
60	GSE17721_LPS_VS_CPG_2H_BMDM_UP	24
61	GSE17721_CPG_VS_GARDIQUIMOD_24H_BMDM_DN	23
62	GSE9988_ANTI_TREM1_VS_LPS_MONOCYTE_DN	23
63	GSE1448_CTRL_VS_ANTI_VALPHA2_DP_THYMOCYTE_DN	23
64	GSE9988_LPS_VS_CTRL_TREATED_MONOCYTE_UP	22
65	GSE22886_CD4_TCELL_VS_BCELL_NAIVE_DN	22
66	GSE27786_NKTCELL_VS_ERYTHROBLAST_DN	22
67	GSE29615_CTRL_VS_LAIV_FLU_VACCINE_PBMC_UP	22
68	GSE29617_CTRL_VS_TIV_FLU_VACCINE_PBMC_2008_UP	21
69	GSE13484_12H_UNSTIM_VS_YF17D_VACCINE_STIM_PBMC_DN	21
70	GSE360_L_DONOVANI_VS_L_MAJOR_DC_DN	21
71	GSE22886_NAIVE_CD4_TCELL_VS_NEUTROPHIL_DN	21
72	GSE2706_UNSTIM_VS_8H_LPS_AND_R848_DC_DN	21

73	GSE18791_UNSTIM_VS_NEWCATSLE_VIRUS_DC_10H_DN	20
74	GSE9988_LOW_LPS_VS_VEHICLE_TREATED_MONOCYTE_UP	20
75	GSE360_HIGH_DOSE_B_MALAYI_VS_M_TUBERCULOSIS_DC_DN	20
76	GSE22886_NAIVE_CD8_TCELL_VS_NEUTROPHIL_DN	20
77	GSE9988_ANTI_TREM1_VS_ANTI_TREM1_AND_LPS_MONOCYTE_DN	20
78	GSE17721_0.5H_VS_24H_CPG_BMDM_DN	20
79	GSE7460_FOXP3_MUT_VS_WT_ACT_TCONV_UP	20
80	GSE14000_UNSTIM_VS_4H_LPS_DC_UP	19
81	GSE1460_NAIVE_CD4_TCELL_CORD_BLOOD_VS_THYMIC_STROMAL_CELL_DN	19
82	GSE17721_POLYIC_VS_CPG_0.5H_BMDM_DN	19
83	GSE360_L_MAJOR_VS_B_MALAYI_LOW_DOSE_DC_UP	19
84	GSE9650_EFFECTOR_VS_EXHAUSTED_CD8_TCELL_DN	18
85	GSE17580_TREG_VS_TEFF_S_MANSONI_INF_UP	18
86	GSE2706_UNSTIM_VS_8H_R848_DC_DN	18
87	GSE26928_EFF_MEMORY_VS_CXCR5_POS_CD4_TCELL_DN	17
88	GSE13306_RA_VS_UNTREATED_TCONV_DN	17
89	GSE2706_UNSTIM_VS_2H_R848_DC_DN	17
90	GSE17721_CTRL_VS_CPG_4H_BMDM_DN	17
91	GSE17974_CTRL_VS_ACT_IL4_AND_ANTI_IL12_1H_CD4_TCELL_DN	17
92	GSE3982_BASOPHIL_VS_NKCELL_UP	16
93	GSE2706_R848_VS_LPS_2H_STIM_DC_DN	16
94	GSE32423_CTRL_VS_IL7_MEMORY_CD8_TCELL_DN	16
95	GSE13306_TREG_VS_TCONV_LAMINA_PROPRIA_UP	16
96	GSE339_EX_VIVO_VS_IN_CULTURE_CD4POS_DC_DN	16
97	GSE9650_NAIVE_VS_EXHAUSTED_CD8_TCELL_DN	16
98	GSE9988_LPS_VS_VEHICLE_TREATED_MONOCYTE_UP	15
99	GSE11924_TH2_VS_TH17_CD4_TCELL_UP	15
100	GSE360_L_MAJOR_VS_B_MALAYI_HIGH_DOSE_MAC_UP	15
	Immunology Signature	
	Tumor Associated	
Rank	GeneSet	p_Normal/ p_Tumor
1	GSE17721_POLYIC_VS_PAM3CSK4_2H_BMDM_UP	422
2	GSE37416_CTRL_VS_6H_F_TULARENSIS_LVS_NEUTROPHIL_UP	111
3	GSE27786_LIN_NEG_VS_ERYTHROBLAST_UP	59
4	GSE27786_CD4_TCELL_VS_NKCELL_DN	56
5	GSE17721_POLYIC_VS_GARDIQUIMOD_4H_BMDM_DN	56
6	GSE17721_CTRL_VS_CPG_0.5H_BMDM_UP	53
7	GSE360_HIGH_DOSE_B_MALAYI_VS_M_TUBERCULOSIS_DC_UP	50

8	GSE11864_UNTREATED_VS_CSF1_IFNG_PAM3CYS_IN_MAC_UP	49
9	GSE29617_DAY3_VS_DAY7_TIV_FLU_VACCINE_PBMC_2008_DN	49
10	GSE17721_POLYIC_VS_GARDIQUIMOD_6H_BMDM_DN	48
11	GSE17721_CTRL_VS_PAM3CSK4_4H_BMDM_DN	43
12	GSE17721_POLYIC_VS_CPG_2H_BMDM_UP	39
13	GSE17974_0.5H_VS_72H_UNTREATED_IN_VITRO_CD4_TCELL_UP	36
14	GSE360_CTRL_VS_M_TUBERCULOSIS_DC_UP	35
15	GSE17721_CTRL_VS_PAM3CSK4_12H_BMDM_UP	34
16	GSE17721_POLYIC_VS_PAM3CSK4_16H_BMDM_UP	31
17	GSE17721_POLYIC_VS_PAM3CSK4_6H_BMDM_DN	30
18	GSE1432_1H_VS_6H_IFNG_MICROGLIA_UP	28
19	GSE14769_UNSTIM_VS_240MIN_LPS_BMDM_UP	25
20	GSE17721_CPG_VS_GARDIQUIMOD_4H_BMDM_DN	24
21	GSE360_L_DONOVANI_VS_M_TUBERCULOSIS_DC_UP	24
22	GSE27786_LIN_NEG_VS_NEUTROPHIL_DN	23
23	GSE360_LOW_DOSE_B_MALAYI_VS_M_TUBERCULOSIS_DC_UP	23
24	GSE17721_CTRL_VS_GARDIQUIMOD_1H_BMDM_UP	23
25	GSE36392_TYPE_2_MYELOID_VS_EOSINOPHIL_IL25_TREATED_LUNG_UP	22
26	GSE17721_CTRL_VS_PAM3CSK4_2H_BMDM_UP	21
27	GSE3982_EOSINOPHIL_VS_CENT_MEMORY_CD4_TCELL_DN	20
28	GSE17721_CTRL_VS_PAM3CSK4_8H_BMDM_DN	20
29	GSE13485_CTRL_VS_DAY21_YF17D_VACCINE_PBMC_UP	20
30	GSE2826_XID_VS_BTK_KO_BCELL_UP	20
31	GSE17721_0.5H_VS_12H_CPG_BMDM_UP	19
32	GSE339_CD8POS_VS_CD4CD8DN_DC_IN_CULTURE_DN	18
33	GSE32423_IL7_VS_IL4_MEMORY_CD8_TCELL_UP	17
34	GSE7852_THYMUS_VS_FAT_TCONV_UP	17
35	GSE27786_NKTCELL_VS_MONO_MAC_DN	17
36	GSE20366_EX_VIVO_VS_HOMEOSTATIC_CONVERSION_NAIVE_CD4_TCELL_UP	16
37	GSE17721_LPS_VS_GARDIQUIMOD_6H_BMDM_DN	16
38	GSE14308_TH17_VS_NAIVE_CD4_TCELL_DN	16
39	GSE17721_CTRL_VS_POLYIC_4H_BMDM_UP	16
40	GSE2826_WT_VS_XID_BCELL_UP	16
41	GSE14308_TH2_VS_NATURAL_TREG_DN	15
42	GSE1460_DP_VS_CD4_THYMOCYTE_UP	15
43	GSE17721_LPS_VS_PAM3CSK4_8H_BMDM_DN	15
44	GSE17721_CTRL_VS_GARDIQUIMOD_6H_BMDM_UP	15
45	GSE17721_LPS_VS_PAM3CSK4_2H_BMDM_DN	14
46	GSE14308_TH2_VS_TH1_DN	14

47	GSE29618_PRE_VS_DAY7_POST_TIV_FLU_VACCINE_BCELL_DN	14
48	GSE32423_IL7_VS_IL7_IL4_MEMORY_CD8_TCELL_UP	14
49	GSE12845_IGD_NEG_BLOOD_VS_NAIVE_TONSIL_BCELL_DN	13
50	GSE3982_MAC_VS_BASOPHIL_UP	13
51	GSE8384_CTRL_VS_B_ABORTUS_4H_MAC_CELL_LINE_UP	13
52	GSE32423_MEMORY_VS_NAIVE_CD8_TCELL_IL7_IL4_DN	13
53	GSE29617_CTRL_VS_DAY3_TIV_FLU_VACCINE_PBMC_2008_UP	13
54	GSE17721_PAM3CSK4_VS_GADIQUIMOD_2H_BMDM_DN	12
55	GSE15324_NAIVE_VS_ACTIVATED_ELF4_KO_CD8_TCELL_DN	12
56	GSE11924_TFH_VS_TH1_CD4_TCELL_UP	12
57	GSE17721_0.5H_VS_4H_CPG_BMDM_UP	12
58	GSE10094_LCMV_VS_LISTERIA_IND_EFF_CD4_TCELL_UP	12
59	GSE14308_NAIVE_CD4_TCELL_VS_INDUCED_TREG_DN	12
60	GSE11864_UNTREATED_VS_CSF1_IFNG_PAM3CYS_IN_MAC_DN	11
61	GSE27786_CD4_TCELL_VS_MONO_MAC_UP	11
62	GSE9650_NAIVE_VS_EFF_CD8_TCELL_UP	11
63	GSE17974_IL4_AND_ANTI_IL12_VS_UNTREATED_0.5H_ACT_CD4_TCELL_UP	11
64	GSE37416_0H_VS_3H_F_TULARENSIS_LVS_NEUTROPHIL_DN	11
65	GSE14769_40MIN_VS_360MIN_LPS_BMDM_UP	11
66	GSE29618_PRE_VS_DAY7_POST_LAIV_FLU_VACCINE_PDC_DN	11
67	GSE39820_TGFBETA1_VS_TGFBETA3_IN_IL6_TREATED_CD4_TCELL_UP	11
68	GSE7764_IL15_NK_CELL_24H_VS_SPLENOCYTE_DN	11
69	GSE17974_0.5H_VS_72H_IL4_AND_ANTI_IL12_ACT_CD4_TCELL_UP	11
70	GSE360_L_DONOVANI_VS_B_MALAYI_HIGH_DOSE_DC_DN	10
71	GSE3982_MEMORY_CD4_TCELL_VS_TH2_DN	10
72	GSE2197_IMMUNOSUPPRESSIVE_DNA__VS_UNTREATEDIN_DC_DN	10
73	GSE3982_DC_VS_NEUTROPHIL_UP	10
74	GSE17721_0.5H_VS_24H_GARDIQUIMOD_BMDM_DN	10
75	GSE1460_DP_THYMOCYTE_VS_NAIVE_CD4_TCELL_CORD_BLOOD_UP	10
76	GSE17974_0H_VS_72H_IN_VITRO_ACT_CD4_TCELL_DN	10
77	GSE12845_NAIVE_VS_DARKZONE_GC_TONSIL_BCELL_DN	10
78	GSE37416_0H_VS_3H_F_TULARENSIS_LVS_NEUTROPHIL_UP	10
79	GSE27786_CD8_TCELL_VS_ERYTHROBLAST_UP	10
80	GSE17721_CTRL_VS_GARDIQUIMOD_2H_BMDM_UP	10
81	GSE12845_IGD_NEG_BLOOD_VS_PRE_GC_TONSIL_BCELL_DN	10
82	GSE9037_CTRL_VS_LPS_1H_STIM_IRAK4_KO_BMDM_UP	10
83	GSE15750_DAY6_VS_DAY10_EFF_CD8_TCELL_UP	10
84	GSE10239_NAIVE_VS_KLRG1HIGH_EFF_CD8_TCELL_DN	9

85	GSE360_CTRL_VS_T_GONDII_MAC_UP	9
86	GSE15930_STIM_VS_STIM_AND_IFNAB_72H_CD8_T_CELL_DN	9
87	GSE360_CTRL_VS_L_DONOVANI_DC_UP	9
88	GSE29618_LAIV_VS_TIV_FLU_VACCINE_DAY7_BCELL_DN	9
89	GSE15659_CD45RA_NEG_CD4_TCELL_VS_ACTIVATED_TREG_UP	9
90	GSE3982_CENT_MEMORY_CD4_TCELL_VS_TH2_DN	9
91	GSE31082_DP_VS_CD4_SP_THYMOCYTE_UP	9
92	GSE29618_PRE_VS_DAY7_POST_LAIV_FLU_VACCINE_MONOCYTE_DN	9
93	GSE1448_ANTI_VALPHA2_VS_VBETA5_DP_THYMOCYTE_DN	9
94	GSE6269_FLU_VS_STREP_AUREUS_INF_PBMC_UP	9
95	GSE339_EX_VIVO_VS_IN_CULTURE_CD4POS_DC_UP	8
96	GSE17580_TREG_VS_TEFF_UP	8
97	GSE37416_CTRL_VS_12H_F_TULARENSIS_LVS_NEUTROPHIL_UP	8
98	GSE17721_LPS_VS_CPG_8H_BMDM_DN	8
99	GSE27786_LSK_VS_CD4_TCELL_UP	8
100	GSE14769_40MIN_VS_360MIN_LPS_BMDM_DN	8

Table S6. Top 100 gene set signatures differentially associated with survival. The gene sets are defined either as ‘normal associated’ (more associated with survival using normal data) or ‘tumor associated’ (more associated with survival using tumor data). The gene set signatures are from the oncogenic signatures and immunology signatures defined by MSigDB.
